# Reply to: ‘Hiding in plain sight’ and ‘Caution is needed when communicating analyses based on an apple to orange comparison’

**DOI:** 10.1093/hropen/hoad017

**Published:** 2023-05-15

**Authors:** Pedro Melo, Simon Wood, Georgios Petsas, Yealin Chung, Julija Gorodeckaja, Malcolm J Price, Arri Coomarasamy

**Affiliations:** Tommy’s National Centre for Miscarriage Research, Institute of Metabolism and Systems Research, College of Medical and Dental Sciences, University of Birmingham, Edgbaston, UK; CARE Fertility Birmingham, Edgbaston, UK; CARE Fertility Chester, Chester, UK; CARE Fertility Sheffield, Sheffield, UK; Tommy’s National Centre for Miscarriage Research, Institute of Metabolism and Systems Research, College of Medical and Dental Sciences, University of Birmingham, Edgbaston, UK; CARE Fertility Birmingham, Edgbaston, UK; CARE Fertility London, London, UK; Institute of Applied Health Research, University of Birmingham, Edgbaston, UK; NIHR Birmingham Biomedical Research Centre, University Hospitals Birmingham NHS Foundation Trust and University of Birmingham, Birmingham, UK; Tommy’s National Centre for Miscarriage Research, Institute of Metabolism and Systems Research, College of Medical and Dental Sciences, University of Birmingham, Edgbaston, UK; CARE Fertility Birmingham, Edgbaston, UK

Sir,

We thank Pirtea *et al.* (2023) and Alsbjerg and Humaidan (2023) for their interest in our article and have addressed below the points we deemed pertinent.

In our original review (Melo et al., 2021), we found a wide range of serum progesterone thresholds below which live birth or ongoing pregnancy rates appeared to be diminished in frozen embryo treatment (FET). The evidence was heterogeneous, even among articles reporting exclusively on cycles using vaginal progesterone for luteal phase support. It is important to note that in that publication, rather than suggesting that serum progesterone measurements ≥10 ng/ml were the level for which to strive, we simply reported that among studies investigating cut-offs lower than 10 ng/ml (which ranged from 8.06 to 9.43 ng/ml), higher serum progesterone levels than the specified threshold were associated with improved outcomes (Melo et al., 2021). There is a subtle yet crucial difference between these two statements. Rounding it up to 10 ng/ml would have simplistically ignored the cut-off heterogeneity we identified in our review.

Our most recent study attempted to investigate what happens in an unselected population of participants presenting for FET, irrespective of (but adjusting for) cycle regimen (Melo et al., 2022). Overall, for serum progesterone levels lower than 7.8 ng/ml, the evidence identified a reduction in live birth rates, which was consistent with the findings of our systematic review. Our article does not suggest that 7.8 ng/ml should be the revised cut-off. Instead, we state that our findings support the aforementioned evidence for thresholds lower than 10 ng/ml. Where our analyses differed from much of the existing evidence was in the way we evaluated serum progesterone as a continuous variable, thus minimizing loss of data and statistical power (Altman and Royston, 2006). We discussed extensively the limitations of our cohort in our article, including its pragmatic nature, which we tackled by adjusting our analyses for clinically important confounders, the variation in sample timing, and the difficulties in reaching the sample size originally planned (Melo et al., 2022). We agree with Alsbjerg and Humaidan (2023) that the low sample size resulted in imprecision, warranting further studies on this important topic, particularly in non-vaginal routes of progesterone administration.

Our study is not unique in identifying evidence of a non-linear association between serum progesterone and treatment outcomes in FET. In the first published cohort on this topic, Yovich et al. (2015) found an optimum range of serum progesterone in FET cycles using vaginal progesterone (70–99 nmol/l or 22–31.1 ng/ml), above which there was a decline in live birth rate. Similarly, Kofinas et al. (2015) showed that in women receiving intramuscular progesterone for luteal phase support in FET, participants with serum progesterone levels above 20 ng/ml exhibited lower rates of ongoing pregnancy or live birth and an increased rate of miscarriage. Alsbjerg et al. (2020) later demonstrated that in FET cycles primed with vaginal and rectal progesterone, serum progesterone levels above 14 ng/ml appeared to negatively affect treatment outcomes. More recently, in a prospective study evaluating combined vaginal and intramuscular progesterone in FET, Alyasin et al. (2021) identified a reduction in live birth rate for serum progesterone levels above 32.5 ng/ml.

The aforementioned studies varied in their FET regimens and found a wide range of serum progesterone thresholds (14–32.5 ng/ml) above which there may have been a negative association with live birth rate. In our own study, we demonstrated that serum progesterone levels below the 10^th^ centile were overall associated with a reduction in live birth. However, we also observed a decrease in live birth rates in women exclusively receiving subcutaneous progesterone who exhibited higher serum measurements. In this subgroup, our adjusted analysis suggested that progesterone levels above 16.3 ng/ml may have been associated with a reduction in effectiveness, which contrasted with the shape of the relationship between serum progesterone and live birth for all other cycle regimens. We acknowledge this finding differs from some of the existing literature—but not all, as demonstrated above. Further, we believe that constructing an argument based on publications from 40 years ago with sample sizes smaller than ours and focusing on average serum progesterone levels in interventional studies, does little to advance the scientific discussion on this topic. This is particularly important within the realm of serum hormone measurements, for which the overwhelming scientific consensus is that optimum ranges exist. It could be argued that suggesting progesterone would be unique by exhibiting no upper serum limit of effectiveness and safety is in fact what truly defies ‘biological plausibility’. Given the conflicting evidence in the available literature, further research examining this specific relationship would be important.

We described our findings in a balanced and well-thought-out manner, including all subgroups of FET regimens in the box aimed for patients. Suggesting we focused exclusively on a potential negative effect of higher serum in women receiving solely subcutaneous progesterone is a misrepresentation of our words, which we chose carefully. We also note that our statement that ‘…in women receiving only injectable progesterone, higher progesterone levels may have reduced the chance of a live birth’ is very different to asserting that ‘this regimen can harm FET implantation’. We regret that Pirtea *et al.* (2023) misquoted us when describing the basis for their letter. In essence, our findings highlight that the fundamental uncertainty about this topic remains, and further mechanistic and clinical data are warranted. Finally, we believe that our results should instigate additional well-designed and appropriately powered clinical studies to further investigate how higher-order progesterone levels in cycle regimens using non-vaginal routes, alone or in combination, may affect FET outcomes. Where single studies are insufficiently powered to confidently answer this question, we suggest that individual patient data meta-analysis should be undertaken.
